# Desmoplastic Small Round Cell Tumor, a “Floating Island” Pattern in Pleural Fluid Cytology: A Case Report and Review of the Literature

**DOI:** 10.1155/2015/676894

**Published:** 2015-08-27

**Authors:** Hui Zhu, Emily Marie McMeekin, Charles D. Sturgis

**Affiliations:** Pathology and Laboratory Medicine Institute, Cleveland Clinic, 9500 Euclid Avenue, Cleveland, OH 44195, USA

## Abstract

Desmoplastic small round cell tumor (DSRCT) is a rare aggressive sarcoma with characteristic clinical and pathologic features. It typically involves pelvic and abdominal organs of young male patients, and patients usually present at advanced stage with poor prognosis. A few reports are available describing the cytopathologic features of DSRCT in serous effusions, with the majority of published cases depicting undifferentiated small blue cells that need to be distinguished from other small blue cell tumors. We report an interesting case of DSRCT involving a pleural effusion with a “floating island” pattern that has been described in hepatocellular carcinoma, renal cell carcinoma, and adrenal cortical carcinoma. In our case, the epithelioid tumor cells form cohesive aggregates surrounded by a single layer of spindle cells, mimicking the “endothelial wrapping” in other tumors with “floating island” patterns. We demonstrate, by ancillary testing, that these peripheral spindle cells are tapered/flattened DSRCT cells, in contrast to endothelial wrapping cells, as seen in other tumors with this unique cytomorphology. To our knowledge, this is the first case report describing DSRCT showing a “floating island” pattern that needs to be differentiated from metastatic hepatocellular carcinoma, renal cell carcinoma, and adrenal cortical carcinoma in effusion cytology.

## 1. Introduction

Desmoplastic small round cell tumors (DSRCTs) are aggressive malignancies with characteristic clinical presentations, pathological findings, and specific associated chromosomal translocations t(11;22) (p13;q12) involving EWSR1 and WT1 genes. These tumors most commonly present with pelvic and abdominal organ involvement in male patients less than 40 years of age. DSRCTs usually present at advanced stages, are resistant to treatment, and pursue aggressive clinical courses with average survival of 3 years or less [1]. Histopathologic findings are specific. The tumors present as nests or cords of undifferentiated small blue tumor cells surrounded by dense fibrous stroma. Immunohistochemically, the tumor cells express cytokeratins and EMA (>90% of cases), desmin (90% of cases), and variable neural markers [2, 3]. The characteristic translocations [t(11;22)] with resultant EWSR1-WT1 fusions can be detected by fluorescence in situ hybridization (FISH) or reverse transcriptase-polymerase chain reaction (RT-PCR) technology. Confirmatory cytogenetic and/or molecular testing is often pursued, as DSRCTs are rare malignancies and are often associated with poor clinical outcomes.

Cells of metastatic sarcoma are rarely diagnosed in cytologic preparations of serous effusions, and involvement of effusion fluid by DSRCT is extremely uncommon, with fewer than 15 cases reported in the literature. The largest case series of DSRCT involving fluids contained seven cases from five patients [4]. All reported fluid cytology cases of DSRCT describe characteristic morphology of DSRCT including dyshesive small blue cells with high nuclear/cytoplasmic ratios, scant to moderate cytoplasm, granular chromatin, and at least moderate nuclear atypia with frequent molding and mitotic activity. Dense fibrous stromal fragments may be present and are an important clue to the correct diagnosis. In the majority of cases, in the absence of fibrous stroma, the cytomorphologic features of DSRCT are difficult to distinguish from other small round blue cell tumors such as lymphoma, neuroblastoma, Ewing's sarcoma, primitive neuroectodermal tumor (PNET), and small cell carcinoma. Most patients with serous effusion involvement will have a known history of DSRCT. Immunocytochemical studies confirming characteristic cytokeratin and desmin positivity and molecular/genetic testing confirming a characteristic t(11;22) (p13;q12) translocation will prove useful in challenging cases.

In this study we report a case of DSRCT involving a pleural effusion with the lesional cells appearing in “floating island” patterns. The groups of lesional cells are comprised of cohesive, epithelioid tumor cells with moderately abundant cytoplasm and slight nuclear atypia. In some areas, the peripheries of the cohesive cell groups have a thin and incomplete layer of adherent flattened/spindled cells. Similar “floating island” cytomorphologies have been described in cytology specimens obtained from primary and metastatic hepatocellular carcinomas, adrenal cortical carcinomas, and renal cell carcinomas, in which tumor cell nests may be intimately associated with investing outer single layers of endothelial cells [5, 6]. We demonstrate, by immunohistochemical and special staining methods, that the spindle cells at the peripheries of the “floating islands” of DSRCT in serous effusions appear more likely to be spindled forms of the neoplastic cells proper, rather than investing types of nonneoplastic endothelial or mesenchymal cells.

## 2. Case Presentation

**Figure 1:**
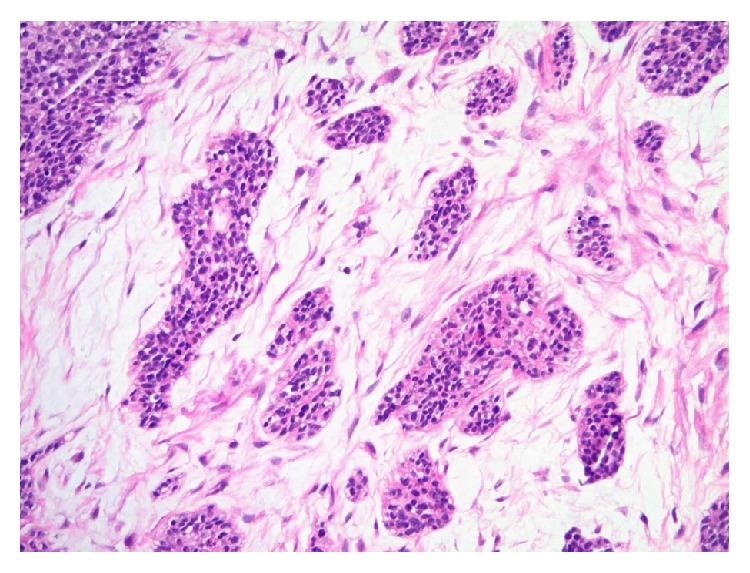
The excised supraclavicular lymph node showed the characteristic morphology of DSRCT. Cohesive nests of undifferentiated small blue cells with some degree of nuclear atypia were seen embedded within moderately cellular fibrous stroma (histopathology, hematoxylin and eosin stain, 100x).

**Figure 2:**
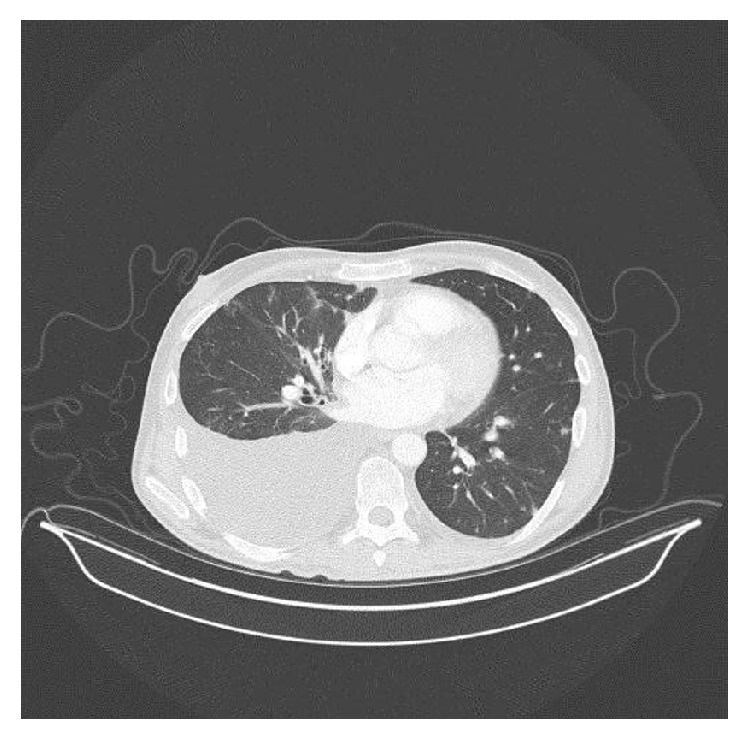
CT examination confirmed a unilateral right pleural effusion and small lung nodules (transverse computed tomographic image of patient's chest demonstrating posterior right chest effusion).

Three years prior to the development of the current thoracic effusion, a 35-year-old man with a past medical history of hypertension, hydronephrosis, kidney stones, and desmoid tumor of the knee presented with fatigue, weakness, an unintentional 14-kilogram weight loss, and left supraclavicular lymphadenopathy. Excisional biopsy of a left supraclavicular lymph node showed replacement of the node by small round cells with intervening dense fibrous stroma (Figure 1). Immunohistochemical studies performed at an outside hospital showed that the tumor cells were positive for AE1/AE3, CK8, CAM 5.2, and CD99. The lesional cells were negative for desmin, EMA, and S-100. Germ cell markers including PLAP, HCG, and AFP were also negative. Markers of neuroendocrine differentiation including chromogranin and synaptophysin were negative, and lymphocytic markers including CD45, CD30, CD3, CD5, CD20, CD138, BCL2, and BCL6 were also nonreactive. The case was then sent to Cleveland Clinic for an expert soft tissue tumor second opinion consultation, as the immunopattern did not exactly fit what was expected for DSRCT by the referring pathologist. The clinical presentation and histologic findings were felt to be diagnostic of DSRCT by two experienced soft tissue pathologist consultants at our center, and the consultants noted that, like all malignant neoplasms, not all examples of DSRCT show “classical” patterns of immunoreactivity. The expert consultants at Cleveland Clinic pursued FISH for the EWSR1 gene (22q12) break-apart, which was positive, confirming the diagnosis of metastatic DSRCT. A definitive diagnosis of DSRCT was rendered, and the patient was managed based upon this diagnosis. Staging by CT scan showed extensive intra-abdominal disease (including liver lesions) and mediastinal lymphadenopathy. With the bulk of the disease being present in the mesentery, an abdominal primary site was favored. The patient was treated with seven cycles of combination chemotherapy (including cyclophosphamide, doxorubicin, ifosfamide, and etoposide). He experienced a partial radiologic and clinical response with diminution of liver, abdominal, and mediastinal lesions. After combination chemotherapy, it was noted that not all of his abdominal lesions could be surgically resected, so he began second-line combination chemotherapy including vincristine and cyclophosphamide. He tolerated the second-line treatment well with stable disease for 21 months, until he was found to have disease progression in the liver and began pazopanib (oral multityrosine kinase inhibitor) therapy. He did well for another 14 months on pazopanib until he presented with bowel microperforation. Biopsy of a mesenteric nodule confirmed persistent abdominal DSRCT. Subsequent CT examination demonstrated progressive disease involving the liver, peritoneum, and abdominal lymph nodes. In addition, enlarged supradiaphragmatic lymph nodes, multiple small bilateral lungs nodules, and a right pleural effusion were noted (Figure 2). Thoracentesis was performed, and the collected right pleural fluid was sent to cytology for morphologic examination as well as to microbiology for exclusion of an infectious etiology. The microbiologic studies of the pleural fluid proved negative. After empiric antibiotic treatment for the bowel microperforation, the patient was hospitalized to begin salvage chemotherapy with etoposide and ifosfamide.

**Figure 3:**
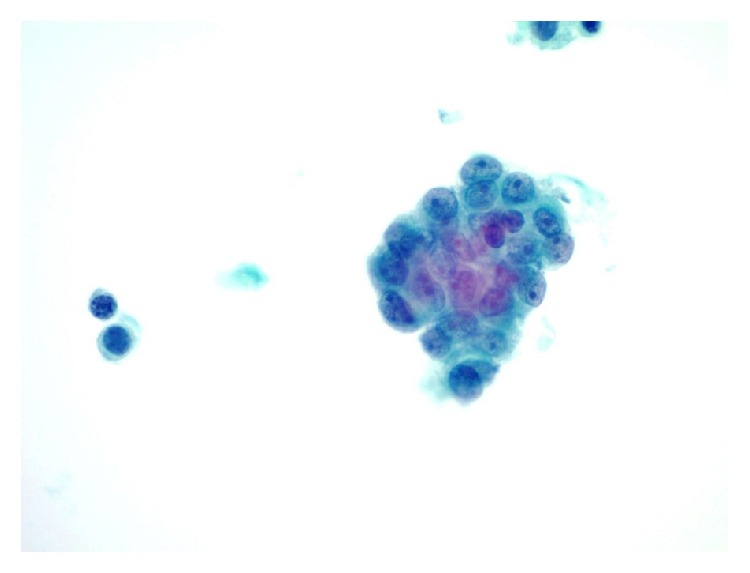
Liquid-based cytology slides contained rare crowded groups of lesional cells with nuclear overlapping, high nuclear/cytoplasmic ratios, open chromatin, and small but discernible nucleoli. These cells were difficult to differentiate from reactive mesothelial cells by microscopy (ThinPrep, Papanicolaou stain, 600x).

**Figure 4:**
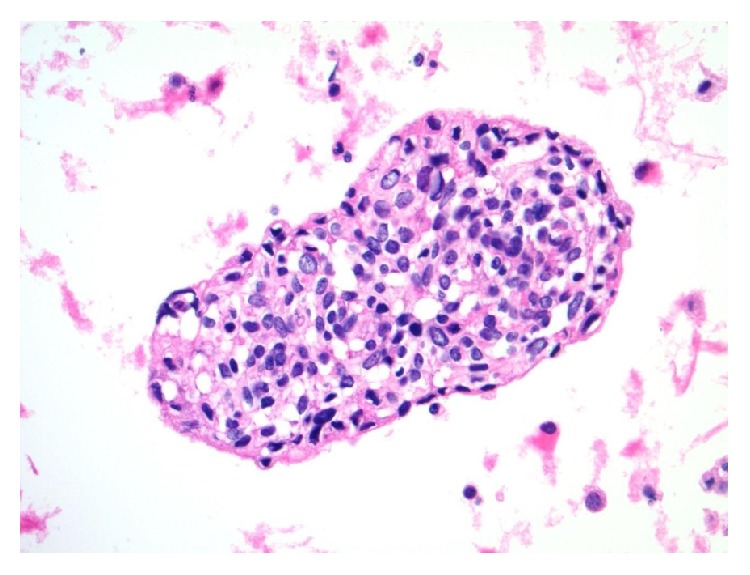
A cohesive and crowded group of metastatic DSRCT cells in pleural fluid with central epithelioid forms having minimal to moderate eosinophilic cytoplasm and open chromatin. Some cells at the tumor group margin have elongated/spindled morphologies with more dense chromatin (cell block, hematoxylin and eosin stain, 400x).

**Figure 5:**
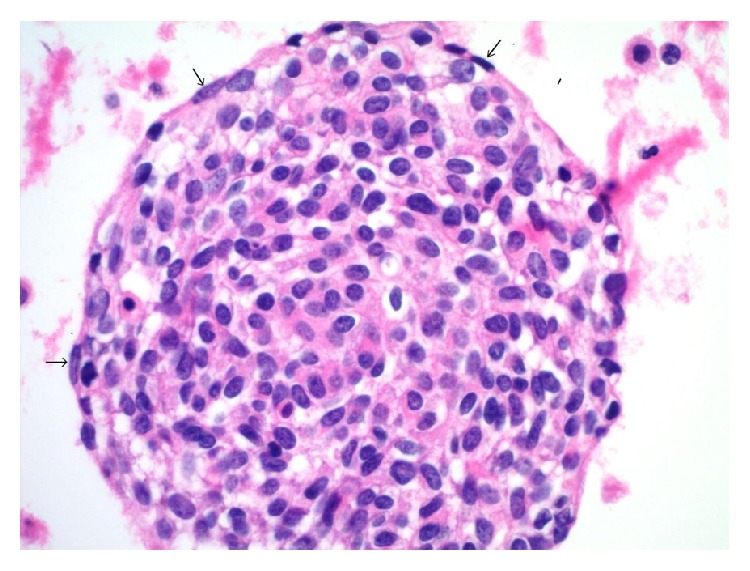
A higher magnification view of DSRCT involving fluid. The image shows a cohesive and crowded group of lesional cells from the pleural fluid with central epithelioid forms. Small arrows mark peripheral elongated/spindled forms. Some of these cells have more dense chromatin, while others have chromatin that is similar to that seen in the epithelioid cells within the body of the island (cell block, hematoxylin and eosin stain, 600x).

The cytology sample (1,000 mL of transparent, nonturbid, yellow fluid) was accessioned and routinely processed with an aliquot of fluid used to create Papanicolaou stained ThinPrep slides. An additional portion of the body fluid was used to create a Cellient cell block that was fixed in 10% neutral buffered formalin and yielded hematoxylin and eosin stained histologic sections. The ThinPrep slides of the pleural fluid showed predominantly a mixture of reactive mesothelial cells, macrophages, lymphocytes, and a few neutrophils. Rare groups of lesional cells were also noted (Figure 3). These groups of cells were cohesive with some degree of cell crowding and nuclear overlapping. An experienced cytotechnologist interpreted these rare lesional cell groups as reactive mesothelial cells. Hematoxylin and eosin stained sections prepared from the cell block showed cohesive groupings of metastatic tumor cells with a “floating island” morphology (Figures 4 and 5). The more central and epithelioid-appearing tumor cells in these groups had bland oval nuclei that were about the same size as mesothelial cell nuclei. The cytoplasm of the lesional cells was minimally to moderately abundant, eosinophilic, and focally vacuolated. Mitoses were infrequent to absent. While no fibrous stromal cell groups were identified in the liquid-based cytology or cell block slides, some of the cell groups in the cell block showed an incomplete single layer of bland elongated/spindled cells intimately applied to the periphery of the tumor cell aggregates. In some areas these peripheral spindled cells were hyperchromatic with dense chromatin, while, in other areas, the nuclear chromatin of these more elongated and less epithelioid cells was better preserved and more similar in tinctorial appearance to that seen in the nuclei of the cells within the intrinsic regions of the cohesive lesional cell groups.

Because of the patient's well-documented history of DSRCT and the availability of histologic materials for cytohistologic correlation, the effusion sample was diagnosed as metastatic DSRCT without a need for ancillary immunohistochemistry or molecular testing.

## 3. Discussion

We report a case of DSRCT in a serous effusion with tumor cells showing “floating island” patterns. Only a few case reports and small case series pertaining to DSRCT involving serous effusions have been published [4, 7–11]. Various morphologic features have been set forth as being of value in diagnosing DSRCT in serous fluids. The majority of existing reports emphasize the presence of lesional undifferentiated small blue cells that are difficult to distinguish from other small blue cell tumors such as lymphomas, small cell carcinomas, neuroblastomas, and other blastemal neoplasms of adolescence and young adulthood. Some citations have also raised the differential diagnostic consideration that prominent cytoplasmic vacuoles and cohesion in cases of DSRCT involving serous effusions may mimic metastatic adenocarcinoma [4, 12, 13]. To our knowledge, our report is the first in the literature in which a case of DSRCT has been recognized to display a “floating island” pattern that might raise the differential diagnosis of metastatic hepatocellular carcinoma, adrenal cortical carcinoma, or possibly renal cell carcinoma.

**Figure 6:**
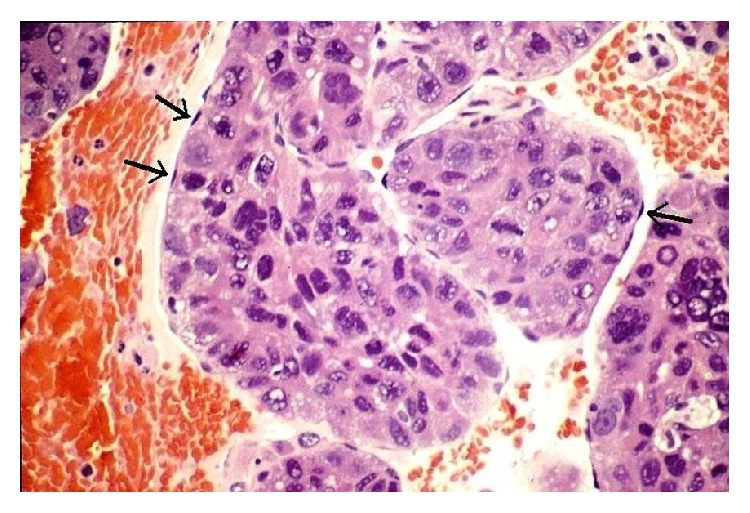
The “floating island” pattern of hepatocellular carcinoma. This image is used to illustrate the unique tumoral growth pattern of cohesive sheets of lesional epithelial cells that are intimately invested by a delicate outer layer of flattened endothelial cells. Arrows mark some of the peripheral wrapping endothelial cell nuclei (cell block, hematoxylin and eosin stain, 400x). This photomicrograph comes from a metastatic moderately differentiated hepatocellular carcinoma in a 64-year-old female and is not from the index patient in the current case report. It is presented for comparison as an example of a morphologic differential diagnosis with some overlapping features with the current unique example of metastatic DSRCT.

**Figure 7:**
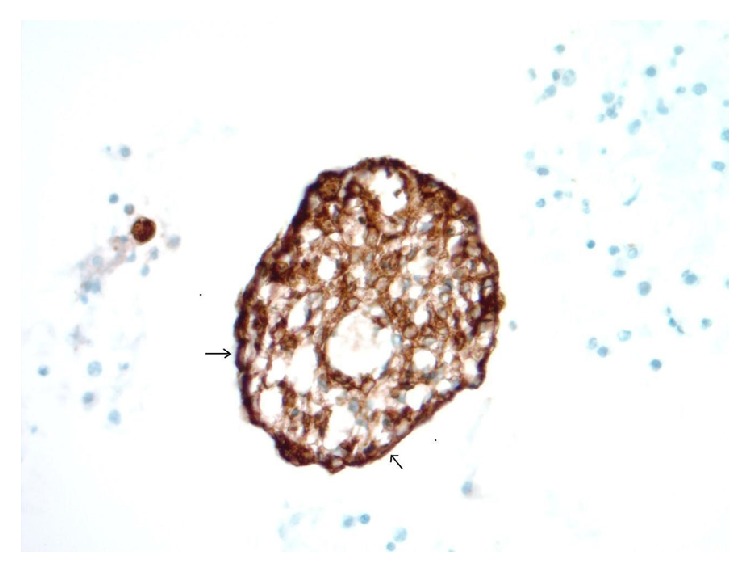
A cohesive group of metastatic DSRCT cells in pleural fluid with central epithelioid forms and a few flattened/elongated cell forms at the periphery of the island, marked with small arrows. The lesional central epithelioid cells are diffusely immunoreactive with cytokeratin AE1/AE3, and the peripheral spindled forms show the same pattern of reactivity with strong cytoplasmic staining (cell block, AE1/AE3 immunohistochemistry, 400x).

**Figure 8:**
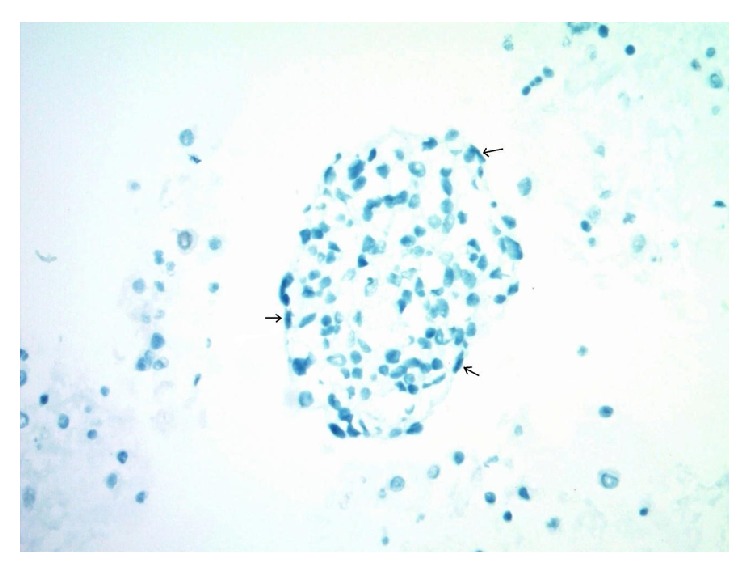
A cohesive group of metastatic DSRCT cells in pleural fluid with central epithelioid forms and a few flattened/elongated cell forms at the periphery of the island, marked by small arrows. The lesional epithelioid cells are nonreactive with smooth muscle actin (SMA), and the peripheral spindled forms show the same pattern of reactivity with no cytoplasmic staining (cell block, SMA immunohistochemistry, 400x).

The moniker of “floating islands” has been previously applied by authors such as Bibbo and de Boer to cohesive aggregates of lesional epithelioid cells surrounded by delicate layers of spindled cells [5, 6]. In cytologic preparations of primary and metastatic adrenal cortical carcinomas, hepatocellular carcinomas, and some renal cell carcinomas, this morphology correlates to cohesive groups of lesional epithelial cells rimmed by variably complete, delicate, outer endothelial wrapping (Figure 6). To our knowledge, an outer incomplete layer of tapered cells has not been previously described in “floating islands” of DSRCTs involving fluids. Ancillary testing confirmed these peripheral spindle cell forms to have the same patterns of cell block immunoreactivity as the epithelioid central cells in the DSRCT cell groups. The peripheral flattened cells were immunoreactive with cytokeratin AE1/AE3 and were negative for smooth muscle actin (Figures 7 and 8). In addition, a trichrome stain performed on the cell block showed no adherent mesenchymal cells. Based upon light microscopy and results of ancillary testing, we conclude that the partially ringing spindled cells in the “floating islands” of metastatic DSRCT represent tapered/flattened malignant cells (tumor cells proper), in contrast to the endothelial wrapping documented by others in adrenal cortical carcinomas and hepatocellular carcinomas.

EWSR1 rearrangements have been documented in many soft tissue neoplasms, including Ewing's sarcoma/primitive neuroectodermal tumor (EW/PNET), DSRCT, low grade myoid tumor, myxoid liposarcoma, extraskeletal myxoid chondrosarcoma, sclerosing epithelioid fibrosarcoma, myoepithelial tumor, angiomatoid fibrous histiocytoma, clear cell sarcoma of soft tissue, clear cell sarcoma-like tumor of gastrointestinal tract, and primary pulmonary myxoid sarcoma. Each of these soft tissue tumors with EWSR1 rearrangements is associated with characteristic clinical presentations and histopathologic findings. The only EWSR1 rearranged tumor that is likely to show overlapping clinical presentation and immunohistochemical features with DSRCT is EW/PNET. However, EW/PNET and DSRCT have differing histologic findings. EW/PNET cases usually show monotonous tumor cells with fine chromatin and extensive necrosis, while DSRCT cases may demonstrate more pleomorphism. In addition, dense fibrous stroma is a characteristic intrinsic feature of DSRCT, but not EW/PNET. By combining clinical setting, disease presentation, histologic/cytologic features, immunohistochemistry, and fluorescence in situ hybridization studies (using dual color, break-apart probes for the EWSR1 gene) it is possible to specifically diagnose DSRCT and to exclude EW/PNET, as was historically done in this case [2].

DSRCTs are aggressive sarcomas with characteristic clinical and pathologic features. These tumors typically involve pelvic and abdominal organs of young patients and show a male predominance [2]. Patients usually present at advanced stage, and prognoses are generally poor. Curative drug therapies are not available, and the best treatments seem to be complete surgical resections, when possible [1]. Cases of DSRCT typically harbor the unique t(11;22) (p13;q12) translocation, which may be confirmed by FISH and/or PCR studies. While sarcomas rarely involve serous cavities, DSRCT has been described in peritoneal fluid cytology cases [14]. The number of cases in the literature is small (fewer than 15 patients described, to our knowledge). It is possible that chemotherapy may change the morphology of tumors with resultant therapy related atypias and degenerative changes being previously reported [15]. Cytologic studies in the current case showed most of the lesional cell groups to be present in the cell block slides rather than the liquid-based cytology preparations, and these studies revealed the unique appearance of “floating island” patterns of lesional cells. (One speculation as to why the ThinPrep slide showed fewer lesional cell groups than the cell block may be that ThinPrep processing includes a proprietary filtering step, and the comparatively large sized “floating islands” of tumor in the fluid may be one explanation for lower cellularity in the liquid-based slide.)

Recognition of islands of cohesive small lesional cells with minimal to moderate cytoplasm and some vacuolization with patchy peripheral spindled forms may be of value in establishing a correct diagnosis and/or in formulating appropriate differential diagnoses and selecting ancillary testing. The flattened cells at the periphery of the “floating islands” in DSRCT appear to be true tumor cells and not investing mesenchymal or endothelial wrapping cells.
